# Complex Permittivity Measurements of Steel Fiber-Reinforced Cementitious Composites Using a Free-Space Reflection Method with a Focused Beam Lens Horn Antenna

**DOI:** 10.3390/s21237789

**Published:** 2021-11-23

**Authors:** Kyeongyong Cho, Sunghui Jo, Yeong-Hoon Noh, Namkon Lee, Sungwook Kim, Jong-Gwan Yook

**Affiliations:** 1 Department of Electrical and Electronic Engineering, Yonsei University, 50 Yonsei-ro, Seodaemun-gu, Seoul 03722, Korea; ky.cho2018@yonsei.ac.kr (K.C.); shjo@yonsei.ac.kr (S.J.); yh.noh@yonsei.ac.kr (Y.-H.N.); 2 Department of Infrastructure Safety Research, Korea Institute of Civil Engineering and Building Technology, 283 Goyangdae-ro, Ilsanseo-gu, Goyang-si 10223, Korea; nklee@kict.re.kr (N.L.); swkim@kict.re.kr (S.K.)

**Keywords:** steel fiber-reinforced concrete (SFRC), focused-beam lens horn antenna, free-space measurement, reflection method, complex permittivity, wave impedance

## Abstract

To measure the electromagnetic properties of steel fiber-reinforced concrete (SFRC) in the X-band, 1-port measurements were performed using a lens horn antenna in a free-space measurement system. Free-space 1-port calibration with translations of the position of the reflector regarding the characteristics of the focused beam lens horn antenna was applied. The intrinsic impedance and complex permittivity of the SFRC were obtained from the measured reflection characteristics. The steel fiber content increased and the electromagnetic properties of the SFRC gradually changed from a dielectric to a conductor, even in very low frequencies compared to the plasma frequencies of general metal, which are optical frequencies. This is considered to be the plasmon effect of the metallic structure formed by the steel fiber. This result is applicable for analyses of the electromagnetic phenomenon of large structures with fiber content.

## 1. Introduction

Steel fiber-reinforced concrete (SFRC) has been used as a building material because it exhibits improved mechanical properties compared to conventional concrete [1]. The non-destructive testing techniques with microwaves are widely used to examine mechanical properties, the degree of curing, the cracks of building materials, and so on. These methods have a low cost, are relatively easy to use, and consume a low amount of power [2,3,4]. There are also studies using the microwave method to examine the distribution of fiber distributions [5,6]. Although steel fibers are added to strengthen the mechanical properties of concrete, they also have electrical conductivity, which provides the potential to generate intentional electromagnetic phenomena, such as shielding, absorbing, reflecting, and so on [7,8]. When a significant amount of electromagnetic waves is reflected due to the inclusion of steel fibers, the transmission characteristic becomes small, and a shielding room is required to accurately measure it. In this study, instead of measuring both the transmission and reflection characteristics, a method that can measure the dielectric constant by measuring only reflection characteristics was applied to solve the problem.

Concrete with sufficient curing and drying is considered to require a mixture of various types of dielectric materials. The equivalent relative permittivity of typical concrete varies depending on the composition of the mixture such as cement and aggregate and is known to have a value of approximately 5–10 in microwave frequencies. Meanwhile, the imaginary part of the relative permittivity, which determines the attenuation of the electromagnetic wave propagating inside the concrete, has a value of 1 or less [9]. When a conductive material is added in the dielectric, the effect of the electrical conductivity of these conductive inclusions appears as an imaginary part of the effective permittivity of the mixture [10]. Reinforced concrete with rebar exhibits an electromagnetic shielding effect at low frequencies and has a large-scale wire-mesh structure [11]. In the case of the SFRC, the electromagnetic properties are changed by the electrically small steel fiber. As the content of the steel fiber increases, the properties of the entire mixed concrete become more likely to be conductive; as a result, the reflection increases and the transmission decreases [12].

Permittivity and permeability are fundamental material properties that determine the behavior of electromagnetic waves in a material. The electrical conductivity can be regarded as an imaginary part of the permittivity. There are several measurement methods to determine these properties. One of the methods involves measuring resistance, capacitance, and inductance by making a material a component of the circuit, which is applicable in DC or low frequencies under 100 MHz. To measure the permittivity and permeability in microwave frequencies, the reflection method, the reflection/transmission method, and the resonator method are used [13]. The procedure of the reflection/transmission method is explained as follows. First, a sample is placed between two-port transmission lines, and the reflection and transmission characteristics of the electromagnetic wave for the sample are measured. The permittivity and permeability are then determined. The Nicolson–Ross–Weir (NRW) method is a well-known method for extracting permittivity and permeability from the measured S-parameter using a 2-port system [14]. Several studies have been conducted to estimate the accurate permittivity based on this method [15]. This method can conveniently measure the material characteristics in a wide frequency range but requires a thin and planar sample. Moreover, if the magnitude of reflection and transmission are around 1 or 0, respectively, it is challenging to estimate the permittivity and permeability simultaneously. In this case, the impedance is the only parameter that can be measured accurately. If the sample does not have magnetic properties, the permittivity can be determined from the impedance.

To determine the homogeneity condition of the mixture, the sample must be sufficiently large compared to the inclusion particles. The length of the steel fiber used in this study was 19.5 mm, and it was contiguous to 30 mm, which is the wavelength of the frequency at 10 GHz. The thickness of the sample used for the reflection/transmission method was close to the half-wavelength. It should be noted that there is no guarantee that the distribution of the direction of the fiber is isotropic. The size of the sample should be sufficiently large to ensure homogeneity and effective and accurate measurements. However, this is not comparable to the condition of the sample thickness for reflection/transmission methods. As the thickness of the SFRC sample increases, the transmission of electromagnetic waves through the sample becomes small. Considering all of these situations, we measured only the reflection from, and the impedance of, the SFRC sample [16]. The conversion method of the reflection-only method in this case is similar to the method of the NRW-based transmission/reflection method.

In this study, detailed steps to measure the impedance of the SFRC sample are discussed. The 1-port calibration process is processed in the vector network analyzer and the free-space measurement system. The time-gating process is additionally conducted to eliminate the multi-reflections from the external environments. The free-space method was used to examine the effective characteristics of rectangular concrete blocks. The frequencies of the measurements are within the X-band range (from 8.2 to 12.4 GHz). The focused beam horn antenna was selected to minimize the effects of the experimental circumstances [17]. The 1-port calibration method for the focused beam environment with reflectors in several positions was proposed to improve the accuracy of the measured reflections. The conversion method for reflection was applied to examine the transmission characteristics of the SFRC samples. The results of the measurement are as follows. The magnitude of the reflection coefficient increases, while the phase decreases as the fiber content increases. The impedance exhibits characteristics opposite to those of the reflections. Note that, with an increase in the steel fiber, the imaginary part of the permittivity is increased, and the real part of the permittivity is decreased, which sometimes even changes to a negative value, which is considered to be due to the plasmon effect. The electromagnetic properties of the SFRC are changed from dielectric to conductor as the fiber content increases, even at a very low frequency compared to the plasma frequency of the general metal. It is believed that the metallic structure was formed with the increase in fiber content. The 1-port reflection calibration method with the focused beam lens horn antenna and the result of the complex permittivity of the SFRC in the X-band are the contributions of this article.

## 2. Methodology

### 2.1. 1-Port Calibration Methods

**Figure 1:**
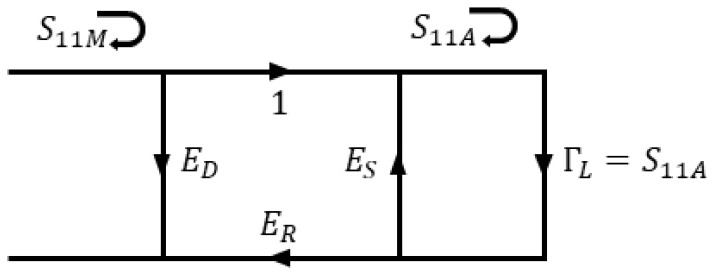
Signal flow graph of the 1-port measurement system.

When a measurement of S-parameters representing the transmitted and reflected signals from the device or material under test through a vector network analyzer (VNA) is conducted, various effects in the space where the signal travels from the port of the VNA to the location of the material specimen surface are included. Phase delay and distortion occur as waves propagate through these regions. These effects include the loss of cables and connectors through which signals pass and reflections due to impedance mismatch. In the case of free-space measurement, the characteristics of the $S_{11}$ of the antenna and the reflections and noises from unwanted experimental circumstances were added. Figure 1 shows the signal flow graph for the measurement environment for the 1-port VNA system [18], and its parameters are expressed as follows.

(1) $$S_{11M}=E_{D}+E_{R}\frac{S_{11A}}{1-E_{S}S_{11A}},$$

(2) $$S_{11A}=\frac{S_{11M}-E_{D}}{E_{S}\left(S_{11M}-E_{D}\right)+E_{R}},$$

Three error terms, that is, $E_{D}$, $E_{R}$, and $E_{S}$, represent ’Directivity’, ’Reflection tracking’, and ’Source matching’ errors, respectively. To determine these three error terms, three independent experiments, for which the measurement results of $S_{11}$ are well known, were performed. Afterwards, three equations were established from the experiments and solved to obtain the value of $S_{11}$, with only the effect of the reflection characteristic due to the sample. When performing 1-port calibration (in general, VNA measurements), ’Short’, ’Open’, and ’Load’ (or ’Match’) are selected as three known standards, and the reflection characteristics are measured. This method is called the ’SOL’ (Short-Open-Load) or ’OSM’ (Open-Short-Match) calibration method. It is assumed that the true results of each reflection characteristic have values of −1, +1, and 0, respectively, for all frequencies, and the error terms are then calculated. The three equations established in this manner can be derived from (1) or (2) as follows:

(3a) $$S_{11M,Short}=E_{D}-E_{R}\frac{1}{1+E_{S}},$$

(3b) $$S_{11M,Open}=E_{D}+E_{R}\frac{1}{1-E_{S}},$$

(3c) $$S_{11M,Load}=E_{D}$$

**Figure 2:**
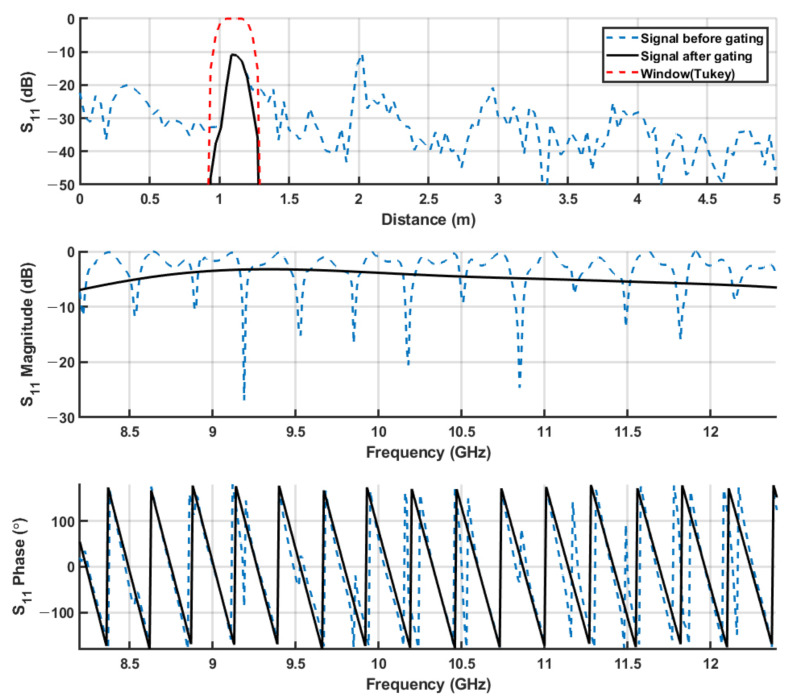
The process of time-gating.

In free-space environments, the multi-reflections from the external environment exist. The time-gating process is conducted to eliminate the multi-reflection components. The process is expressed in Figure 2. First, the target signal in the time domain is selected according to the measurement system. Second, the selected part of the signal is gated with the Tukey window. The gated signal in the frequency domain is much more stable than the non-gated signal.

The measurement of the free space (the empty space with time-gating or absorbing material) can be viewed as ’Load’ or ’Match’, which is the same as $E_{D}$. It can also be viewed as a measurement that represents the $S_{11}$ characteristic of the antenna itself. A flat metal plate made of a good conductor reflects most of the electromagnetic wave energy with a phase transition of $180^{\circ }$; as a result, this metal reflector is considered to be ’Short’. ’Open’ is not realizable in the free-space measurement systems because the reflector needs to be a good magnetic conductor. To replace this ’Open’ condition, a translated metal reflector can be utilized as the third condition. The formula for this is as follows:

(4) $$S_{11M,3^{rd}}=E_{D}+E_{R}\frac{\Gamma_{L}}{1-E_{S}\Gamma_{L}},$$

where $\Gamma_{L}$ is the difference between the reflections from the original metal reflector and those from the translated reflector. This method is called the short-short-load (SSL) calibration method and is generally used in mmWave waveguide measurement systems [19]. In general, $\Gamma_{L}$ in the waveguide measurement has the magnitude of not much less than 1 because of the low conductor loss. However, the magnitude of $\Gamma_{L}$ in free-space measurements varies significantly according to the radiation characteristics of the antenna. The distances of translation and the magnitudes of $\Gamma_{L}$ need to be determined, as explained in the next Section 2.2, considering the characteristics of the focused beam lens horn antenna.

### 2.2. Characteristics of the Focused Beam Lens Horn Antenna

A high-directivity horn antenna is typically used for free-space measurement. For an ideal measurement, a plane wave should reach a flat surface of the sample in the direction of normal incidence; for this far-field radiation condition, there should be a sufficient distance between the sample and the antenna. In addition, the sample size should be sufficiently large to minimize the diffraction occurring at the edge of the sample. To satisfy the far-field condition, as the distance between the sample and the antenna increases, the ratio of the area of the surface of the sample to the beam width of the antenna decreases, and the diffraction effect occurs due to the sample edge. Therefore, it is important to determine an appropriate distance for the typical horn antenna system.

**Figure 3:**
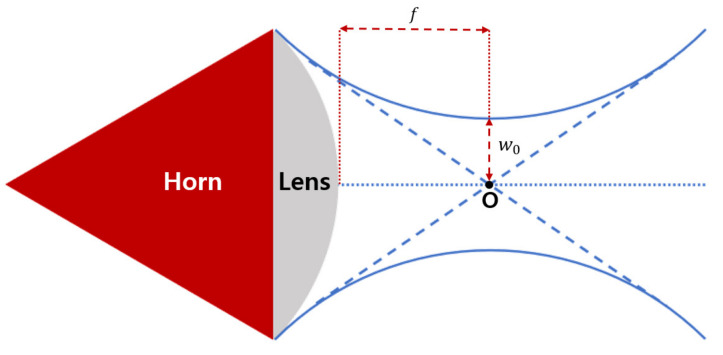
Diagram of the focused-beam lens horn antenna.

**Figure 4:**
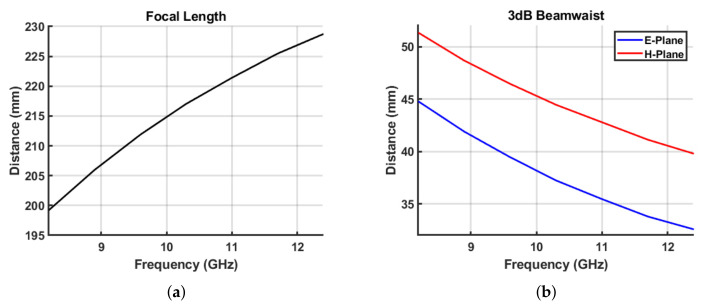
Characteristics of the focused-beam lens horn antenna. (**a**) Focal Length. (**b**) 3 dB Beamwaist.

By placing a dielectric (convex) lens behind the horn antenna, the radiated waves can be concentrated, thereby reducing the required distance for the far-field condition and the effect of diffraction at the edge. Depending on the degree of concentration of the electromagnetic wave, a plane wave is emitted, or the wave is focused as a beam. In the former plane wave case, because the far field is formed at a very short distance, it is easy to control the distance between the antenna and the sample. In the focused-beam case, the measurement is carried out by placing the sample around the focal point, assuming that a Gaussian beam is radiated. The focused beam method has some disadvantages. That is, the results become inaccurate as the sample moves away from the focal point and when the thickness of the sample increases. However, the advantage is that the required surface area of the sample is much smaller. The parameters of the lens antenna that need to be considered are the focal length *f*, which is the minimum distance between the focal point and the lens, and the 3 dB beamwaist $w_{0}$, which is the half width at half maximum of the Gaussian beams at the focal point. Figure 3 shows the diagram and parameters of the focused beam lens horn antenna. *f* and $w_{0}$ are functions of the frequency because the radiation pattern of the antenna and the refractive index of the lens dielectric vary depending on the frequency. In general, as the frequency increases, the focal length increases, and the beamwaist decreases, as shown in Figure 4 [20].

**Figure 5:**
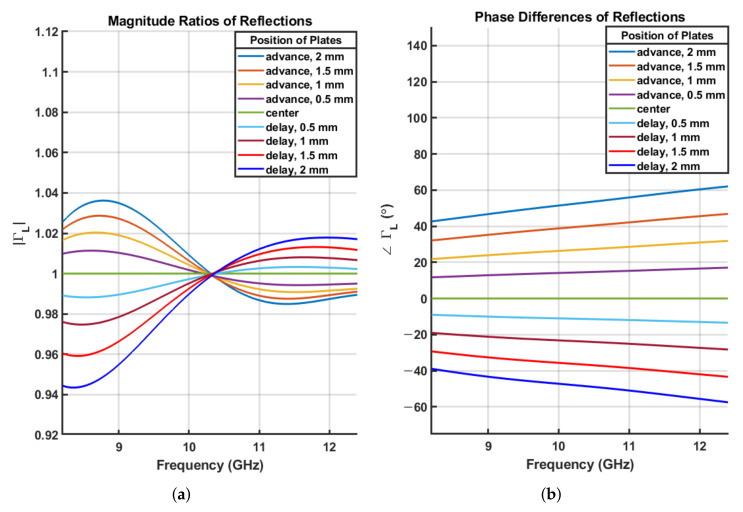
Variation in $\Gamma_{L}$ according to the position of the reflector. (**a**) Magnitude of $\Gamma_{L}$. (**b**) Phase of $\Gamma_{L}$.

Figure 5 shows the variations in $\Gamma_{L}$ according to the positions of the metal plate in ’Short’ stand measurements. The focal point was fixed at the center of the X-band (10.3 GHz). As the metal plate moved from near (advance) to far (delay), the phase of $\Gamma_{L}$ varied in proportion to the distance. In the lower frequency region, the magnitude of $\Gamma_{L}$ is invariant at the center frequency, but it becomes larger at smaller distances and smaller at longer distances from the focal point. The opposite tendency was observed at higher frequencies. The beam is distorted by focusing or spreading as the frequency changes, and the level of the distortion is increased with the distance of the translation. The distortion of the reflection between advance and delay appears to be anti-symmetrical. Using only one advance or delay in the SSL 1-port calibration cannot remove this distortion; however, if both are used with the same distance, the distortion can be countervailed. This process improves the accuracy by at least 1% of the magnitude of the reflection measurements compared to when only one advance or delay is used.

### 2.3. Material Characteristics Conversion Methods

**Figure 6:**
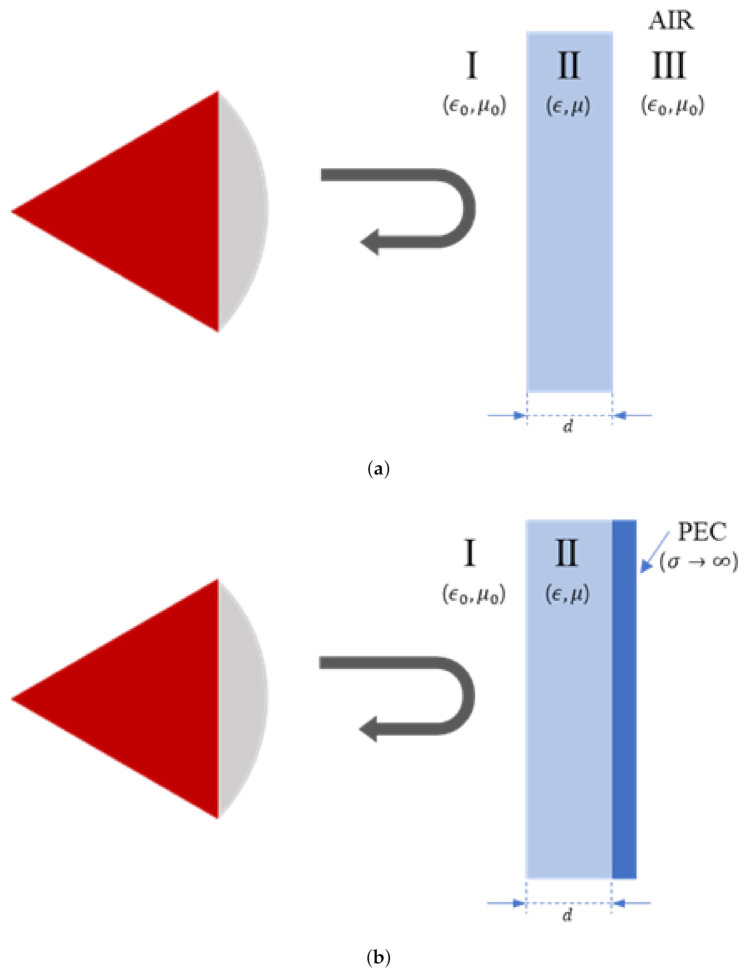
Measurements of two reflection cases. (**a**) Air-back case. (**b**) Metal-back case.

Methods for measuring material properties using reflection properties are reported in [21,22]. In this section, the material property estimation method is presented. The method involves placing two materials with different reflective properties behind the measurement sample and comparing the reflection characteristics of both materials. Two materials, air and a metal plate, were used so that the differences in reflections can be clearly distinguished. The basic concept of this method is similar to the NRW method, in which both the permittivity and permeability are determined from the reflection characteristics. However, as shown in Figure 6, $S_{11}$ values are measured for two different situations, metal-back and air-back, and the relationship between the transmission/reflection coefficients and $S_{11}$ with multiple reflections can be derived for these two situations.

The measured $S_{11}$ values for each case with air and metal are $A_{11}$ and $P_{11}$, respectively. The relationships between them and the reflection coefficient *R* and transmission coefficient *T* are as follows:

(5a) $$A_{11}=\frac{R-RT^{2}}{1-R^{2}T^{2}},$$

(5b) $$P_{11}=\frac{R-T^{2}}{1-RT^{2}}.$$

From Equation (5a,b), *T* and *R* can be obtained. Now, $T^{2}$ is given by (6):

(6) $$T^{2}=\frac{A_{11}-R}{(A_{11}R-1)R}=\frac{P_{11}-R}{P_{11}R-1}.$$

If we rearrange this as a quadratic equation for *R* and then solve it, *T* and *R* can be determined as

(7) $$R=-X\pm \sqrt{X^{2}-1},\;\;where\;\;\;X=\frac{1}{2}\left[1-P_{11}-\left(1+P_{11}\right)A_{11}^{-1}\right]$$

In this case, an appropriate solution of *R* should have a magnitude of less than 1. Moreover, similar to the well-known NRW method, the relative complex permittivity and permeability can be determined using *T* and *R*.

(8a) $$\epsilon_{r}=-\frac{\ln T}{d}\frac{1-R}{1+R},$$

(8b) $$\mu_{r}=-\frac{\ln T}{d}\frac{1+R}{1-R}.$$

Note that, when the magnitude of *R* and *T* is close to 1 and 0, respectively, the magnitude of reflection from the opposite side of the sample to the antenna is too small to measure, and almost identical reflections are measured regardless of the material behind the sample. It can be assumed that the measured $S_{11}$ and the reflection characteristic *R* are the same because the multi-reflection component barely exists when $A_{11}\approx P_{11}$. In this case, the reflections can be determined directly from the calibrated $S_{11}$ (9), and the surface impedance *Z* of the material can be determined using (10), where $Z_{0}={\left(\mu_{0}/\epsilon_{0}\right)}^{0.5}$ is the intrinsic impedance of free space.

(9) $$R\approx S_{11}\;\;\left(where\;T\approx 0\right),$$

(10) $$Z=Z_{0}\frac{1+R}{1-R},$$

When the sample is non-magnetic, the complex permittivity can be determined using (11), assuming that the relative permeability of the material is unity.

(11) $$\epsilon_{r}=\frac{Z_{0}^{2}}{Z^{2}}\;\;\left(where\;\mu_{r}=1\right)$$

## 3. Measurements

**Figure 7:**
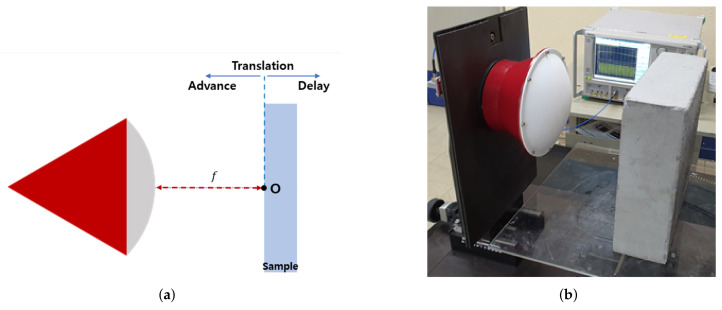
The free-space measurement environment. (**a**) Diagram of the measurement environment. (**b**) Measurement system with concrete sample.

The free-space measurement environment for measuring the reflections from a concrete sample is as follows. In this study, a focused beam lens horn antenna whose band ranges from 8.2 to 12.4 GHz was used, and the S-parameters were measured using the VNA. The gain of the used lens horn antenna is over 30 dB, and a typical $S_{11}$ is −20 dB. The diameter of the lens is 222 mm, and the material of the lens is polytetrafluoroethylene (PTFE). After 1-port SOL calibration using a commercial calibration kit, the reflections from the SFRC were measured by applying additional 1-port SSL calibration in free space, which was above in Section 2.2. An aluminum plate with a size of 300 mm × 300 mm × 3 mm was used as a reflector. The size of the concrete blocks was 300 mm × 300 mm × 100 mm, whose cross section was sufficiently larger than the 3 dB beamwaist of the antenna. Five concrete samples with 0.2%, 0.3%, 0.4%, 0.5%, and 1.0% steel fiber content (volume fraction) were measured. The radius and the length of the steel fiber were 0.1 mm and 19.5 mm, respectively. The concrete blocks had approximately 15,000 fibers per 0.1% fiber content. In this experiment, the measurements were performed at five points on the surface of the SFRC. One is placed at center and the others are spaced at 50 mm above, below, left, and right from the center.Some specimens of the fiber content were manufactured twice and measured; however, since there was no significant difference in the results, only one was produced for the remaining specimens. The diagram and the realized measurement system are shown in Figure 7a,b.

The specimens without steel fiber and 0.1% steel fiber cases were also measured, and no significant differences in the measurement results were observed for $A_{11}$ and $P_{11}$. In this case, multiple reflections can exist, and the assumption of S11≈R is not valid. Thus, it is challenging to accurately measure the impedance. Note that the use of $A_{11}$ and $P_{11}$ to obtain the permeability would be inappropriate in addition to determining whether there are multiple reflections. This is due to the fact that the thickness of the specimen is quite large and that the surface on the opposite side is far out of focus of the lens antenna. Therefore, impedance measurements were performed for specimens containing 0.2% or more steel fibers, which is considered to have a shielding effect of a certain degree by satisfying the condition $A_{11}\approx P_{11}$.

## 4. Results and Discussion

**Figure 8:**
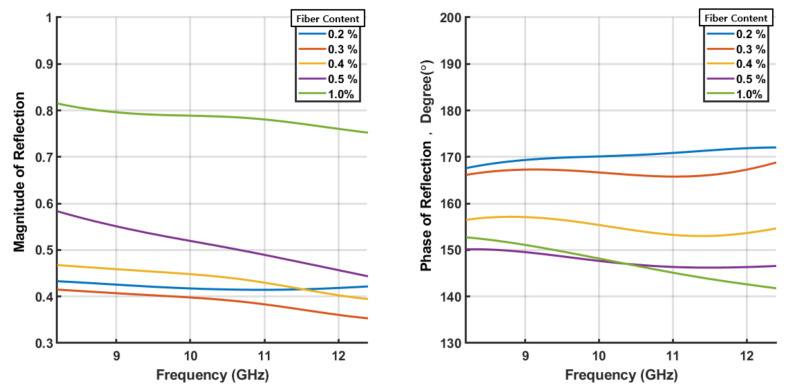
Reflection coefficient of each specimen of the steel fiber reinforced concrete (SFRC).

**Figure 9:**
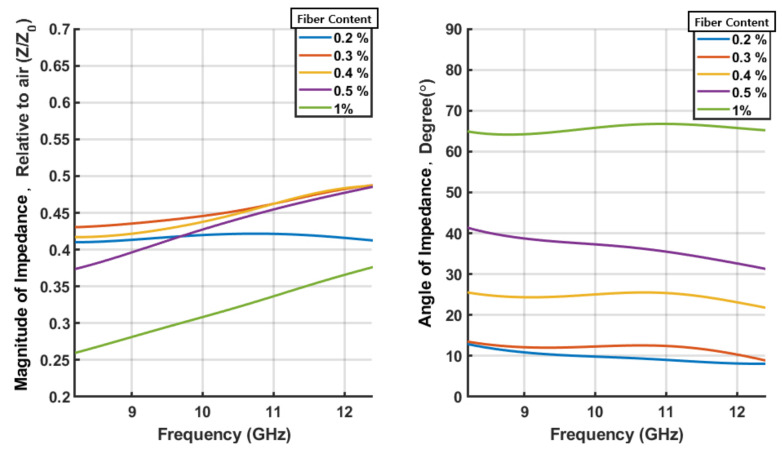
Impedance of each specimen of the SFRC.

**Figure 10:**
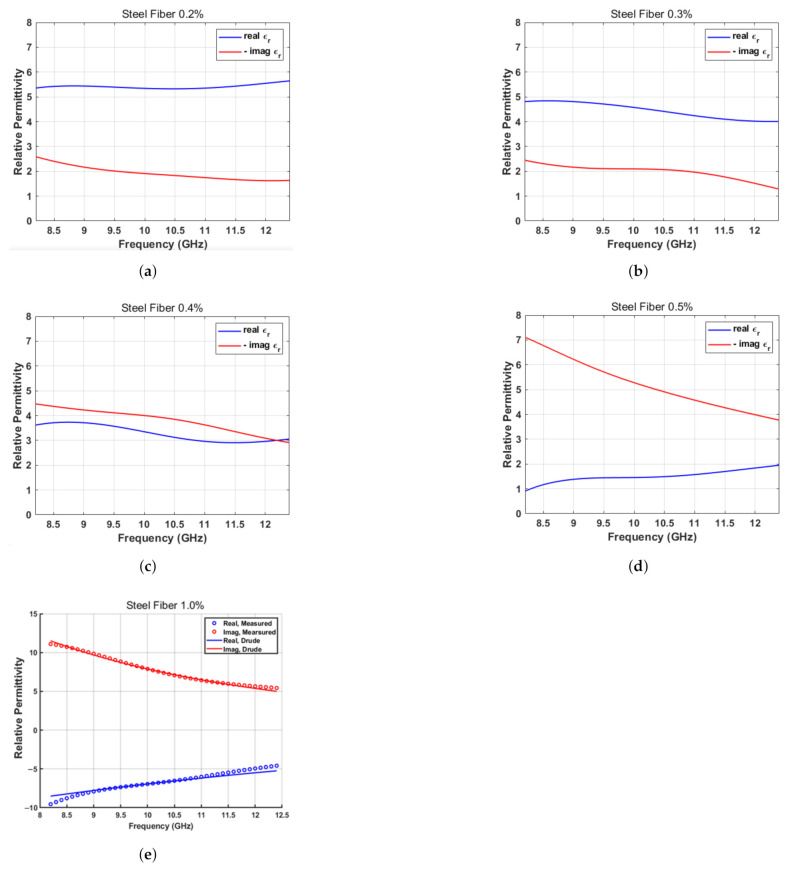
Complex permittivity of each fiber concentration. (**a**) SFRC 0.2%. (**b**) SFRC 0.3%. (**c**) SFRC 0.4%. (**d**) SFRC 0.5%. (**e**) SFRC 1.0%.

The magnitude and phase of the reflection characteristics for the specimens are shown in Figure 8. The magnitude and angle of the impedance for the SFRC samples are calculated as shown in Figure 9. The magnitude of the impedance is expressed as a relative value with respect to the impedance of the free space, $Z_{0}=377\;\Omega$. The relative complex permittivity ($\epsilon_{r}$) calculated from the impedance, assuming that the relative permeability ($\mu_{r}$) of the specimens is unity, is shown in Figure 10 for five different steel fiber concentrations.

**Figure 11:**
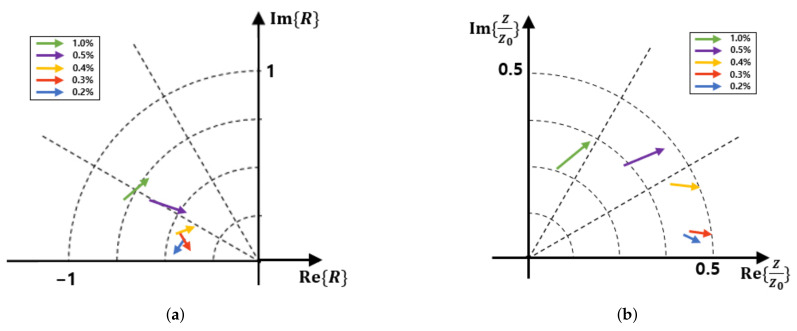
Reflection and impedance in complex planes. (**a**) Reflection. (**b**) Impedance.

It is interesting to note that the magnitude of the reflections increased as the content of the steel fiber increased. The impedances obtained from these reflections decrease in magnitude as the steel fiber content increases. As for the complex permittivity, the real part gradually decreased, whereas the imaginary part increased as the steel fiber content increased. In particular, in the case of the 1.0% steel fiber specimen, the real part of the permittivity is negative. This can be attributed to the change in the properties of the concrete block from those of a dielectric to those of a conductor with the increase in steel fiber content. Figure 11 shows the results plotted on the complex plane. The direction of the arrow indicates an increase in frequency. Note that the relation between the reflection coefficient and impedance is known as the Smith chart.

Table 1 shows the measured values of each specimen at the center frequency 10.3 GHz.

The negative value of the real part of the permittivity in the SFRC with a high steel fiber content (1.0%) can be considered the plasmon effect of the metallic structure at low frequencies [23]. The Drude or Lorentz-oscillation model can be used to explain this result:

(12) $$\epsilon_{r}=1+\frac{\omega_{p}^{2}}{(\omega_{0}^{2}-\omega^{2})+j\omega \gamma }.$$

The least square estimation result of the measured value and that obtained by the Drude model is shown in Figure 10e. The modeled value of the plasma frequency $\omega_{p}$ is 40 GHz, and the resonant frequency $\omega_{0}$ is 0.2 GHz. The random distribution of the steel fiber has self-inductance and results in a plasmon effect.

Notably, there are certain issues regarding the electromagnetic properties of the SFRC that need to be considered. The random distribution of steel fibers may generate some magnetic properties owing to the ferromagnetism of the steel, and an effective ring resonator can also be formed by arbitrary connections of steel fibers. Moreover, the homogeneity of the steel fiber in the concrete block is an important factor in determining the reflections. If the distribution of the location or direction of the fibers is not homogeneous or isotropic, the phase of the reflection and the results of the impedance and permittivity change accordingly.

The 1-port free space measurement system can only measure the reflection characteristics with the absorptive or reflective specimen. If the specimen has a magnetic property, the calculated value of permittivity from the impedance may not be accurate. The impedance is the only material parameter that is correctly measured in this case. The other limitation of the method is that the distance between the antenna and the sample is a sensitive factor that determines the phase of the reflection characteristics, which is important for determining the impedance. The dislocation of a 1 mm result in a phase difference such as $12^{\circ }$ occurs at the frequency 10 GHz, whose wavelength is 30 mm. In order to ensure that the error of the phase of the reflection is less than $1^{\circ }$, an accuracy of distance adjustment within about 0.1 mm in the X-band is required.

## 5. Conclusions

The reflective characteristics of steel fiber-reinforced concrete were measured using the 1-port free-space measurement technique. The accuracy of the reflection characteristics calibrated with reflectors at both the delay and advance was at least 1% compared to when only one additional reflector was used. The impedance and complex permittivity were estimated from these reflective characteristics. As the content of steel fibers increased from 0.2 vol.% to 1.0 vol.%, the magnitude of the reflections increased from 0.4 to 0.8, but the phase of the reflections showed a decrease from a degree of 170 to 150. It was confirmed that the impedance and complex permittivity results exhibited the same trend. The real part of the permittivity decreases from 5.5 to −7, and the imaginary part increases from 2 to 7 at the center frequency of 10.3 GHz. The negative value of the real part of the permittivity in the SFRC with a steel fiber content of 1.0 vol.% shows that, as the content of steel fibers in the concrete increased, the properties of the SFRC changed from those of a dielectric to those of a conductor with plasmon effects.

This study regards a method for determining electromagnetic properties of building materials by measuring reflection properties, when it is difficult to measure the insignificant transmission properties of the specimen. It can be applied to simulations that can aid in the electromagnetic analysis of large structures containing steel fibers. If a database is built for more diverse concrete and steel fibers conditions, conversely, it can be used to determine the distribution or dimensions of the steel fiber in the building materials from the reflection characteristics. Furthermore, it will be possible to proceed with measurement and result analysis for more diverse conductive inclusions such as carbon fiber.

## Figures and Tables

**Table 1:** The result of the measured reflection, impedance, and permittivity of the SFRC at the center frequency (10.3 GHz).

SF Content	*R*	$Z/Z_{0}$	$\epsilon_{r}$
0.2 vol.%	$0.41∠170^{\circ }$	$0.42∠10^{\circ }$	5.3−j1.9
0.3 vol.%	$0.39∠166^{\circ }$	$0.45∠12^{\circ }$	4.5−j2.1
0.4 vol.%	$0.44∠154^{\circ }$	$0.44∠26^{\circ }$	3.2−j3.9
0.5 vol.%	$0.51∠148^{\circ }$	$0.44∠37^{\circ }$	1.5−j5.0
1.0 vol.%	$0.79∠147^{\circ }$	$0.32∠66^{\circ }$	−6.7−j7.4

## Data Availability

Not applicable.
